# Advantages and Limitations of 16S rRNA Next-Generation Sequencing for Pathogen Identification in the Diagnostic Microbiology Laboratory: Perspectives from a Middle-Income Country

**DOI:** 10.3390/diagnostics10100816

**Published:** 2020-10-14

**Authors:** Nurnabila Syafiqah Muhamad Rizal, Hui-min Neoh, Ramliza Ramli, Petrick @ Ramesh A/L K Periyasamy, Alfizah Hanafiah, Muttaqillah Najihan Abdul Samat, Toh Leong Tan, Kon Ken Wong, Sheila Nathan, Sylvia Chieng, Seow Hoon Saw, Bee Yin Khor

**Affiliations:** 1 UKM Medical Molecular Biology Institute (UMBI), Universiti Kebangsaan Malaysia, Kuala Lumpur 56000, Malaysia; nabilasyafiqah13@gmail.com; 2 Department of Medical Microbiology and Immunology, Faculty of Medicine, Universiti Kebangsaan Malaysia, Kuala Lumpur 56000, Malaysia; ramliza@ppukm.ukm.edu.my (R.R.); alfizah@ppukm.ukm.edu.my (A.H.); muttaqillah@ppukm.ukm.edu.my (M.N.A.S.); wkk@ppukm.ukm.edu.my (K.K.W.); 3 Department of Medicine, Faculty of Medicine, Universiti Kebangsaan Malaysia, Kuala Lumpur 56000, Malaysia; petrick@ppukm.ukm.edu.my; 4 Department of Emergency Medicine, Faculty of Medicine, Universiti Kebangsaan Malaysia, Kuala Lumpur 56000, Malaysia; sebastianttl@yahoo.co.uk; 5 Department of Biological Sciences & Biotechnology, Faculty of Science and Technology, Universiti Kebangsaan Malaysia, Selangor 43600, Malaysia; sheila@ukm.edu.my (S.N.); sylvia@ukm.edu.my (S.C.); 6 Faculty of Science, Universiti Tunku Abdul Rahman, Perak 31900, Malaysia; sawsh@utar.edu.my; 7 BioEasy Sdn. Bhd., Selangor 40170, Malaysia; beeyin@bioeasy.com.my

**Keywords:** bacterial pathogen identification, culture and biochemical testing, 16S rRNA next-generation sequencing, middle-income countries, diagnostic microbiology

## Abstract

Bacterial culture and biochemical testing (CBtest) have been the cornerstone of pathogen identification in the diagnostic microbiology laboratory. With the advent of Sanger sequencing and later, next-generation sequencing, 16S rRNA next-generation sequencing (16SNGS) has been proposed to be a plausible platform for this purpose. Nevertheless, usage of the 16SNGS platform has both advantages and limitations. In addition, transition from the traditional methods of CBtest to 16SNGS requires procurement of costly equipment, timely and sustainable maintenance of these platforms, specific facility infrastructure and technical expertise. All these factors pose a challenge for middle-income countries, more so for countries in the lower middle-income range. In this review, we describe the basis for CBtest and 16SNGS, and discuss the limitations, challenges, advantages and future potential of using 16SNGS for bacterial pathogen identification in diagnostic microbiology laboratories of middle-income countries.

## 1. Introduction

### 1.1. Culture and Biochemical Testing (CBtest) for Bacterial Pathogen Identification

Identification of bacteria that cause infections is important for patient management and transmission control in hospitals. The current gold standard for bacterial pathogen identification in diagnostic microbiology laboratories involves culture and biochemical testing (CBtest); this workflow is widely available in hospitals of most middle-income countries where in-house microbiologists are available [1,2,3]. This includes Malaysia, a middle-income country situated in Southeast Asia. CBtest allows identification of commonly encountered medically important bacteria, and is based principally on bacterial phenotypes such as morphology, colony growth and metabolic features [4]. Gram staining and subsequent microscope observation allow preliminary evaluation of bacteria presumably present in clinical specimens; it is said to be the most useful and inexpensive protocol in the diagnostic microbiology laboratory. Following this, bacteria will be cultured on differential media and allowed to grow, prior to biochemical testing for pathogen identification [5]. It is important that cultured colonies are pure and free from contamination, so that biochemical test results are specific towards the bacteria being tested. Identification of common medically important bacterial pathogens via CBtest can be technically easy and relatively affordable; indeed, standardization of CBtest workflows has led to wide-spread use of this identification method in diagnostic microbiology laboratories of middle- and higher-income countries, including Malaysia [2,3,6].

### 1.2. Limitations of CBtest

One limitation of the CBtest is that not all bacterial species can be successfully cultured. This includes strict anaerobic bacteria which will die in the presence of oxygen if not carefully transported in air-tight containers to the laboratory, viable bacteria that are dormant but non-culturable, and fastidious pathogens which require enriched medium for successful culture [5]. Rare pathogens might escape routine investigation due to the requirement for specific biochemical tests needed for their identification; these tests might not be available in some hospitals in lower and middle-income countries (LMICs) [7,8]. In addition, CBtest results will be obscured if a mixed culture is obtained, as different bacteria species will give different results based on their secreted metabolites [1]. CBtest for bacterial identification is also not ideal if the phenotypic and biochemical profiles of the investigated bacteria change easily due to stress [9]. More importantly, CBtest is time consuming for identification of fastidious or slow-growing pathogens, leading to possible patient morbidity and mortality, broad-spectrum antibiotic usage and the possibility of pathogen transmission to other patients [4,10,11]. This is especially true when infection prevention and control (IPC) strategies are not prioritized, a situation which might occur in LMICs [12,13].

### 1.3. 16S rRNA Next-Generation Sequencing (16SNGS): An Alternative to CBtest

With the advent of Sanger DNA sequencing, bacteria can now be identified via nucleotide sequence of the 16S rRNA gene—a short, conserved gene specific to bacterial genus (96%) and for some, species (87.5%) [10]. This method of bacterial identification is culture-independent and only requires DNA of the tested bacteria [14]. Variable regions of the 16S rRNA gene have also been reported to allow species identification [15]. In addition to its utility in identifying bacterial pathogens from biospecimens obtained directly from patients, the 16S rRNA gene sequencing-based approach is useful in cases of ambiguous CBtest results to avoid potential culture-related biases in pathogen identification.

The development of NGS techniques, including 16S rRNA next-generation sequencing (16SNGS), allows further upscaling of sequencing quantity (fragments versus time) even in mixed cultures, and has been proposed as a possible substitute in place of the CBtest for bacterial identification in diagnostic microbiology laboratories [10,14,16]. Nevertheless, 16SNGS requires procurement of costly equipment, timely sustainable maintenance of these platforms, development of specific laboratory infrastructure and training of technical expertise, all of which are still challenges even for middle-income countries [17,18,19].

### 1.4. Diagnostic Microbiology in Malaysia, a Middle-Income Country

Malaysia, a middle-income country with the third largest economy in Southeast Asia, is poised to achieve high-income country status between 2020–2024 [20,21]. Diagnostic microbiology laboratories in Malaysia are divided into three categories: laboratories located in government hospitals, university laboratories in teaching hospitals, and laboratories in private medical centers or private testing laboratories [6]. These laboratories are located at tier 2 (serving a 50,000–200,000 population) or tier 3 (serving a 3–6 million population) and above hospitals that have in-house microbiologists [2,3]. Decision making and funding for these laboratories are independent in each group, with government laboratories funded by the Ministry of Health, university hospitals by the Ministry of Education, while private laboratories are mostly business entities. At the time of writing, most laboratories are still using CBtest for bacterial pathogen detection, although some laboratories are also conducting nucleic acid-based testing (NAT) [22].

## 2. 16SNGS: Platforms, Workflow and Bioinformatics Analysis

### 2.1. NGS Technology

The era of NGS began with the introduction of pyrosequencing technology by 454 Life Sciences in 2005, followed by the Solexa/Illumina platform, Life Technologies’ SOLiD, Ion Torrent and Ion Proton sequencers, and later, the MiSeq and HiSeq platforms from Illumina [17,23,24]. Even though pyrosequencing is considered the “pioneer” of NGS, it is now no longer available after the platform was discontinued by Roche in 2015. The Illumina and Ion fleet of sequencers operate using a “sequencing by synthesis” chemistry [25], compared to the “sequencing by ligation” technology of the now (also) discontinued SOLiD platform [26]. Due to its ability to read palindromic sequences, “sequencing by synthesis” appears to be the more popular chemistry [27], resulting in the dominance of both Illumina and Ion sequencers (currently: Ion Torrent Genexus System and Ion Gene Studio S5 System) in NGS laboratories. Illumina sequencers are now available in either benchtop or production scale categories, allowing users more options to select the best NGS platform according to their laboratory needs and budget allocations (https://www.illumina.com/systems/sequencing-platforms.html). Of note, the most recent player in the NGS industry will be Complete Genomics (acquired by Beijing Genome Institute (BGI))’s “DNA nanoballs” sequencing platforms (https://en.mgitech.cn/products/) [28]; these platforms were reported to be comparable in performance to Illumina sequencers [29,30,31]. In the recent decade, third-generation or long-read sequencing technologies by Pacific Biosciences and Oxford Nanopore Technology have also been launched [32,33,34], although, due to cost concerns, these platforms are mostly used in research institutes compared to diagnostic laboratories. For a comprehensive overview of NGS platforms and their associated chemistries, readers may refer to reviews by Ambardar et al. [35], Besser et al. [36] and Slatko et al. [37]. Regardless of their sequencing chemistry, all NGS platforms can generate millions of DNA molecules with different yields and sequence lengths via parallel sequencing, and allow simultaneous multiplexing of several hundred samples in a single run [38]. To explore these new technologies, some Malaysian research laboratories such as the Malaysia Genome Institute (MGI) and UKM Medical Molecular Biology Institute (UMBI) procured next-generation sequencers in 2012, but these platforms were only used for research projects and not for clinical diagnosis (Chin Kah Loke, Analisa Resources Sdn. Bhd., personal communication).

### 2.2. 16SNGS Workflow and Bioinformatics Analysis

**Figure 1:**
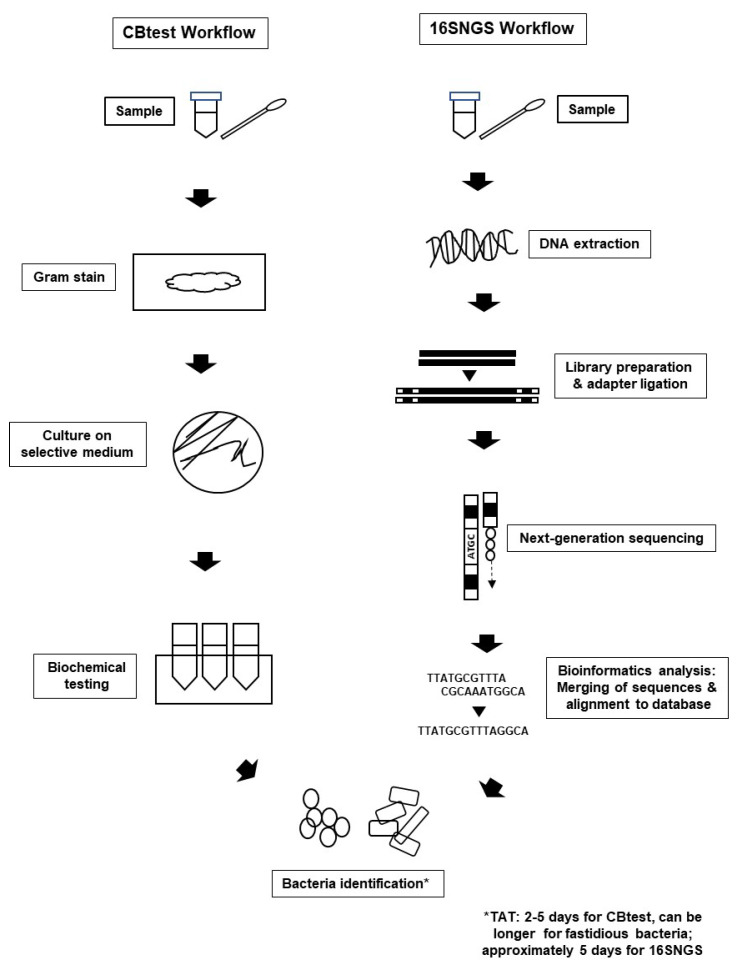
Bacterial identification workflow via CBtest is different from that of 16SNGS. For CBtest, samples are subjected to gram staining and culture on selective medium. Subsequent biochemical testing will reveal the identity of the bacterial pathogen. On the other hand, DNA extraction from samples is carried out in the first step of 16SNGS workflow. After library preparation, NGS of the 16S rRNA fragments will then be done, followed by bioinformatics analysis to identify the infecting bacteria. TAT of the identification process is workflow-dependent. CBtest, culture and biochemical testing. 16SNGS, 16S rRNA NGS. TAT, turn-around time.

Introduction of NGS technologies significantly promoted the development of “metagenomics”. The term “metagenomics” was first used by Handelsman et al. over 20 years ago, where it refers to the study of genetic material from a sample without the need for isolation and culturing of the microorganisms contained in the sample itself [39,40]. Originally used to study microbial community diversity within samples from the environment and also organisms, diagnostic microbiology laboratories were built on the metagenome concept to sequence only the 16S rRNA gene of bacterial populations within clinical specimens to identify infecting pathogens [23,41]. Nevertheless, the workflow for bacterial pathogen identification via 16SNGS is vastly different from that of the CBtest (Figure 1).

Briefly, the 16SNGS workflow starts with genomic DNA extraction of bacteria from biospecimens such as blood, pus, tissue and urine. Genomic DNA is extracted using either conventional protocols or commercialized kits, and subsequently quantified to determine quantity and quality of the extracted DNA [42,43]. Following this, 16S rRNA gene libraries are prepared, from which variable regions of the 16S rRNA gene will be amplified [24,44]. Depending on the sequencing platform, the variable region selected for amplification and sequence for bacterial identification may differ, though it has been reported that the V4-V6 regions are most representative of the full-length 16S rRNA gene [45,46,47,48]. Subsequently, DNA pre-processing to obtain specific sizes of DNA fragments is carried out. Adapters will then be added onto the amplified 16S rRNA region. Following this, quantification and normalization of the amplicons will be carried out prior to sequencing [24,49,50,51].

Increasingly, the processes of DNA extraction and library preparation have been identified as potential bottlenecks of the NGS workflow, especially in a diagnostic laboratory dealing with a large number of samples, daily. To counter this, automated nucleic acid extraction machines, such as the QIAcube (Qiagen Inc.), Maxwell^®^ RSC (Promega Corporation) and KingFisher automated extraction and purification platforms (Thermo Fisher Scientific) allow walk-away DNA extraction [52], and are now an essential component in many large NGS centers. In addition, automation in liquid handling for library preparation is achievable via pipetting workstations such as the Biomek i-Series (Beckman Coulter and Bravo Automated Liquid Handling Platform (Agilent) [53]. In future, microfluidics solutions for NGS library preparation will enable miniaturization and enclosed environment for the process, minimizing contamination and optimizing laboratory space utilization [54,55,56].

After sequencing is completed, raw data pre-processing is important prior to bioinformatics analysis. This includes the screening and removal of sequencing adapters, assessing overall sequencing read quality (quality checking), trimming or filtering low quality reads and filtering of reads based on sequence length. This step is important for removing low quality and erroneous reads. To detect putative chimeric sequences in filtered data, the sequences are normally subjected to chimera check [57]. At this stage, all chimeric sequences are removed before the next step of analysis. For data pre-processing, multiple tools are freely available, including PEAT [58], Trimmomatic [59] and FastQC (Babraham Bioinformatics, Cambridge). For paired-end data, the merging of forward and reverse reads is done as the first step of quality control and could be performed with BBMerge [60]. To analyze the 16S rRNA gene in bacteria, a common approach is via operational taxonomic unit (OTU) clustering, where sequences are clustered into a representative OTU sequence, defined at ≥97% sequence similarity level [61,62]. The OTU-based approach is used to distinguish and differentiate biologically real nucleotide differences from artefacts [63]. The primary output of this approach will be OTU tables represented by BIOM file format. Quantitative Insight into Microbial Ecology (QIIME) [64] is one of the most popular tools for the OTU-based approach. Recently, the amplicon sequence variants (ASVs) approach has been introduced; several pipelines are now available with the aim to correct sequencing errors and improve taxonomic resolution, including DADA2 [65], Qiime2-Deblur [66] and USEARCH-UNOISE3 [67]. Sensitivity and specificity differ between different pipelines, among which DADA2 was reported to have the best sensitivity and resolution. Even though it produces a higher number of spurious ASV compared to others, DADA2 would still be the best choice to obtain the highest possible resolution [68].

Subsequent alignment of the consensus sequences to a reference database will identify the bacteria being investigated. Public repositories of bacterial 16S rRNA gene database are available for this purpose. The NCBI Bacterial 16S Ribosomal RNA RefSeq Targeted Loci Project (https://www.ncbi.nlm.nih.gov/bioproject/33175) curates comprehensive and non-redundant 16S rRNA sequences submitted by the public to the International Nucleotide Sequence Database Collaboration (INSDC) [69]. On the other hand, the Ribosomal Database project hosted by Michigan State University [57] also contains 16S rRNA sequences from INSDC, though it has a smaller source of taxonomy classification compared to NCBI [70]. Pathogen identification can also be done using SILVA (https://www.arb-silva.de), which provides aligned rDNA sequences from Bacteria, Archaea and Eukaryota domains [71]. One interesting attribute of this database is that its curators place emphasis on unculturable environmental bacteria [72], which might be helpful in diagnosis of infections caused by bacteria from the natural environment.

The Greengenes database (http://greengenes.secondgenome.com) is the default database in the QIIME pipeline. Nonetheless, this database is one of the more popular database used in 16SNGS [73]. Greengenes features chimera assessment, allowing the identification of parent sequences which is useful if the diagnostic microbiology laboratory also intends to carry out phylogenetic research for pathogen genomics surveillance. However, the database has not been updated since 2014, and may not contain nomenclature of novel or renamed bacteria after 2014. In recent years, new 16S rRNA gene databases such as the GRD—Genomic-based 16S ribosomal RNA Database (https://metasystems.riken.jp/grd/) and the EzBioCloud 16S database (https://www.ezbiocloud.net/resources/16s_download) have been established. GRD curators correct misannotations or missing anti-SD sites and other short segments of the 16S rRNA gene sequences extracted from complete genomes for more reliable taxonomic assignments. EzBioCloud 16S database is a commercial product; nevertheless, at the time of writing, it is freely available for users from academic and non-profit institutions. The database has been shown to allow bacterial identification to species level and provided taxonomic accuracy comparable to SILVA and Greengenes [74].

## 3. Limitations and Challenges in Implementing 16SNGS for Pathogen Identification in Diagnostic Microbiology Laboratories of Middle-Income Countries

Even though the NGS technique was first initiated around 2005, the workflow has been mainly used for research purposes (such as profiling of environmental bacterial communities and gut microbiome profiling) rather than for pathogen identification in the diagnostic microbiology laboratory [75,76], and this is also the case in Malaysia [77,78,79,80]. Several limitations and challenges towards widespread implementation of the technique for pathogen identification in diagnostic microbiology remain to be overcome, if the platform is to be used in middle-income countries like Malaysia. This includes limitations and challenges in the inherent low taxonomical resolution of 16SNGS sequencing reads, bioinformatics analysis of results, costly laboratory setup and reagents, lack of sample trail for repeat testing and lack of techniques validation.

### 3.1. Low Taxonomical Resolution in 16SNGS Sequencing Reads

The 16S rRNA gene is approximately 1550 bp in length. For 16SNGS, sequencing is usually carried out on one (can be more, but the associated cost will increase) of the variable regions of the gene [48]. Therefore, short sequencing reads (usually spanning about 300–500 bases) from 16SNGS might not be ideal for species resolution of some bacterial genus. Even though short reads from NGS platforms are more accurate, studies comparing the output between NGS and long-read sequencing technologies have shown the latter to produce greater taxonomic classification at the genus and species level [81,82]. In addition, some bacteria may share high similarity with other members of the same family even in the variable regions of their 16S rRNA sequences [83]. For these bacteria, additional sequencing of other genes will lead to more accurate species identification. The *dnaJ* sequence shows superior species identification for *Enterobacteriaceae* compared to 16S rRNA [84]. *Burkholderia* sp. and *Mycobacterium* sp. are better identified using *recA* and the internal transcribed spacer (ITS) region, respectively [85,86]. Mycobacterium species can also be identified using the heat shock protein (*hsp*) sequence [87]. Zeaiter et al. reported using the *ftsZ* sequence for *Bartonella* species resolution [88], while Fournier et al. utilized additional sequences from four genes (*gltA*, *ompA*, *ompB*, *gene D*) in addition to 16S rRNA to identify rickettsia isolates [89]. Of note, Sabat et al. reported improved bacterial species identification via targeted NGS of both 16S and 23S rRNA sequences [43], where the sequencing was performed on DNA extracted directly from urine and orthopedic samples, in addition to those from blood culture bottles. A recent review by Church et al. provides a comprehensive summary on the performance of the 16S rRNA gene sequence for bacterial identification [90].

### 3.2. Bioinformatics Analysis of Results

Data output from 16SNGS is in the form of raw FASTQ reads which require processing and filtering prior to analysis. For this, bioinformatics software packages or online tools such as Trimmomatic, FastQC, PEAR, QIIME 2, MOTHUR are freely available, which will help in resource-limited laboratories [19,91,92]. Nevertheless, usage of these tools usually require knowledge of the Linux platform and text-based command-line such as UNIX, a field where many clinical microbiologists and medical laboratory technicians are neither well-versed nor trained [17]. This gap is also apparent in LMICs [70,93,94]. Bioinformaticians are integral for this purpose—to create pipelines for sequence analysis, as well as for results generation and analysis. While the approach of having a technician-microbiologist-bioinformatician team to conduct sequencing and interpretation of sequence reads together with clinical presentation output might be feasible in a research laboratory, actualization of this pathogen identification process is in many ways impossible for day-to-day workflows in diagnostic laboratories. This is due to the fact that the quantities of samples processed by diagnostic microbiology laboratories are very much larger than research samples, and will create a backlog in the delivery of diagnostic results if bioinformatics analysis are carried out in the conventional manner without any automation.

### 3.3. Costly Laboratory Set-Up, Maintenance, Staff Training and Reagent Procurement

Currently established diagnostic microbiology laboratories in middle-income countries are mostly performing CBtests for pathogen identification whereby the CBtest workflow requires economical and widely available culture media and chemicals. Hence, the transition from CBtest to 16SNGS diagnostics will require substantial funds [17,95,96]. Despite decreasing costs for sequencing, NGS platforms and 16SNGS reagents remain costly while sequencers and their associated accessories such as computer servers require scheduled maintenance.

Laboratory staff, usually only proficient in CBtest, will need training in 16SNGS and bioinformatics workflows. In Malaysia and perhaps other middle-income countries, diagnostic microbiology laboratory technicians might not have received training in molecular biology methods [97]. Therefore, while these technicians are proficient in aseptic techniques, and that CBtest specimens are either from sterile sites or cultured on selective media, most technicians are not aware of the consequence of nucleic acid contamination in 16SNGS workflows. Contaminating bacteria and their DNA could be present or introduced in any step of the sequencing workflow: biospecimen, transport media, reagents and disposables [98]. In addition, laboratory personnel need to avoid cross-contamination of samples and working solutions during the sequencing process, as amplification of even minute concentrations of contaminant DNA could lead to wrong diagnosis for the patient. Closed DNA extraction and library preparation automated systems may reduce episodes of contamination, in addition to laboratory designs with unidirectional workflow and separated pre- and post-amplification stations [99]. However, all these will incur additional costs to the laboratory.

A further limitation is that NGS platforms and reagents might not be readily available in all regions in the world, especially for LMICs, compounding the costs of 16SNGS pathogen detection [19]. Moreover, 16SNGS pathogen detection requires bioinformatics analysis and data storage, which incurs additional costs in the diagnostics pipeline [100]. All the above factors will result in the costs of diagnosis being transferred to the patient if pathogen identification is carried out via 16SNGS and not CBtest. Some patients might not be able to afford costly diagnostics. Ironically, poorer patients, especially low-income communities in middle-income countries, are the ones who generally have a higher risk of getting infections [101].

### 3.4. Lack of Sample Trail for Repeat Testing and Antibiotic Susceptibility Testing (AST)

The starting material for pathogen identification using 16SNGS is DNA; therefore, bacteria cultivation will not be a prerequisite for laboratories intending to use only 16SNGS as its protocol. While this approach might reduce the workload and costs for the laboratory, there might be no sample trail for test repeats when the amount or quality of the extracted test DNA is low and does not pass the required quality control for downstream sequencing [99]. Furthermore, in the current diagnostic microbiology workflow, AST requires pathogen culture. For new hospitals intending to only set up a 16SNGS diagnostic microbiology platform, alternatives for AST have to be considered: either the testing for susceptibility is outsourced to another laboratory (where bacterial culture is carried out), or usage of new and upcoming rapid AST platforms is required (further discussed in this review under Section 5: Future Considerations).

### 3.5. Lack of Workflow Standardization and Validation

The usage of 16SNGS for bacterial pathogen detection is still in active development for many diagnostic microbiology laboratories (including those in high-income countries), and the technique requires standardized protocols and rigorous validation [102,103]. Many 16SNGS workflows remain to be validated, including wet laboratory protocols, data analysis algorithms and reference databases. In addition, every element in the aforementioned workflow also has to be validated, including, and not restricted to, amount of DNA for sequencing, sequencing platform, tools for bioinformatics analyses [103,104]. Quality assurance metrics for the workflow also have to be established [99].

Moreover, due to the huge number of medically important bacterial pathogens, it might not be feasible to validate the identification process for every single pathogen, especially for difficult-to-culture bacteria which cannot be stored. For these cases, in silico proficiency testing has been suggested as an alternative approach, where modified sequences are used for validation of algorithms and sequence database, instead of sequencing output from the bacteria themselves [105]. Nevertheless, laboratory personnel who are competent in in silico proficiency testing remain few for middle-income countries. For many workflows, validation is still actively being carried out by laboratories in high-income countries such as ARUP Laboratories (Salt Lake City, UT, USA), ID by DNA Inc. (Sunnyvale, CA, USA), and the University of California, San Francisco [103], in the United States.

## 4. Advantages of 16SNGS for Bacterial Pathogen Detection

Challenges in setting up 16SNGS platforms for diagnostic microbiology laboratories in middle-income countries are numerous; nevertheless, once solutions are available, the platform offers several advantages compared to CBtest in diagnostic bacteriology.

### 4.1. Identification of Unculturable and Fastidious Bacteria

While it is acknowledged that bacteria cultivation will allow sample trail for re-testing of samples if required, not all bacteria are culturable, or rather, yet culturable [106]. Indeed, environmental microbiologists estimated the frequency of culturable bacteria at merely 2%; while only about 20% of the gut microbiota can be cultured [107,108]. A large proportion of blood cultures (approximately 50%) in clinical practice result in negative identification of the infecting pathogen, either due to the infection being caused by a virus, fastidious growth of the infecting bacteria or initiation of antibiotics prior to blood culture sampling [109,110,111,112]. Anaerobic pathogens might die and result in negative growth if exposed to oxygen during sampling, transport or culture [5]. Fastidious bacteria require specific nutrients to support their growth. Intra-cellular bacteria such as *Rickettsia* spp. and *Coxiella burnetii* require culture systems using embryonated eggs [113] or shell vial cultures [114]; while bacteria such as *Mycobacterium leprae* and some species of *Borrelia* require animal inoculation [115,116]. The need for specific culture systems (“one bug, one test”) might cause a delay or failure in identification of fastidious bacteria via the CBtest, especially for low-resource laboratories and in cases where laboratory technicians lack experience in the culturing of fastidious bacteria [5,18,104]. Usage of 16SNGS for bacterial identification circumvents the need for bacteria cultivation, as the technique only requires DNA of the investigated pathogen. This will enable pathogen identification with straightforward and streamlined protocols from sequencing library generation to bioinformatics analyses.

### 4.2. Shorter and Predictable Turn-Around-Time with Streamlined Identification Protocol

Many medically important bacterial pathogens that are culturable can be identified with a turn-around-time (TAT) of about 3–5 days using CBtest [117,118,119,120] (Rizal et al., manuscript in preparation). Nevertheless, the success of CBtest still hinges heavily on the ability of the diagnostic laboratory to obtain pure cultures [5]. This in turn requires stringent protocols in the whole identification process: sample acquisition, sample transport [121], decontamination of commensal flora [122], use of selective media [123], incubation time [124], temperature [11,125] and atmospheric control [126,127]. For some bacteria such as *C. burnetti* and *Chlamydia* species, axenic media will be essential for identification [5]. This might pose infrastructure and technical difficulties for some laboratories in middle-income countries [8,18].

Variation in the protocols of CBtest workflows to obtain pure cultures usually results in longer TATs for fastidious and slow-growing pathogens [5,114]. In addition, technical knowledge and experience are integral in CBtest, and identification of these pathogens might require dispatching of the samples to reference laboratories and sentinel hospitals which may be located a distance away from the requesting laboratory and hospital [2,127]. This also results in a longer TAT for pathogen identification [114].

For pathogen identification using 16SNGS, differences between protocols are mostly limited to the DNA extraction step, in particular, during bacterial cell wall destruction to release the DNA. For the convenience of its users, many commercially available extraction kits include either universal lysis buffers that lyse a variety of biological specimens containing the bacteria to be identified, or recommend a mechanical lysis procedure to obtain DNA [128,129]. These kits are now available in most middle-income countries, either direct from the manufacturer or from local distributors. The process after DNA extraction until sequencing is similar for all tested bacteria, resulting in standardized TAT for all tests and results generation. Results from our study (in a middle-income country laboratory) consistently show a 16SNGS workflow TAT of 5 days, regardless of the infecting bacteria (Rizal et al., manuscript in preparation). Laboratories with DNA extraction and library preparation automation might be able to achieve even shorter TATs.

### 4.3. Accuracy of Results

Bacterial identification via 16SNGS is based on sequence identity and alignment of the 16S rRNA gene. The 16S rRNA sequences is specific to the level of bacterial genus (and, in some cases, species) [45,46]. On the other hand, CBtest relies on phenotypic identification of tested bacteria via bacterial growth on selective media and bacteria metabolism of various nutrients [1,5]. This approach has been the gold standard in diagnostic microbiology and it undoubtedly resolved the identification of many bacterial pathogens. Nevertheless, biochemical results could be arbitrary and operator-dependent, especially in the circumstances where tested bacteria are not cultured under the correct conditions or amounts to allow release of targeted metabolites for corresponding growth or color change. Furthermore, with polymicrobial infections, results from CBtests will be unspecified, where the test fails to identify one/some of the infecting pathogen(s) [16,130]. This will not be a problem with the 16SNGS protocol, as the 16S rDNA sequence is specific for each bacterial genus. Quality control (QC) procedures ensure accuracy for each 16SNGS workflow; and with the availability of validated genus/species identification bioinformatics pipelines in the future, the 16SNGS platform’s diagnostic accuracy will be very much higher compared to CBtest. This can be related to the fact that, while the phenotypic characteristics of bacteria are considered when establishing nomenclature, confirmation of novel bacteria species is still done via 16S rRNA gene sequencing [131,132]. Some diagnostic microbiology laboratories in Malaysia, Thailand, Nigeria and Kenya have obtained the ISO 15189:2012 Medical Laboratories—Requirements for Quality and Competence accreditation, or have developed in-country standards for CBtest [18,133,134,135]. Malaysia is one of the first middle-income countries to adopt a National Accreditation Scheme for pathology laboratories in December 2004 [2]. Therefore, laboratory personnel in the country are often familiar with the concept of QC and quality assurance in medical testing; this should prepare them for adhering to quality procedures in 16SNGS protocols [99,136]. Of note, patient medical history and subsequent clinical judgment from the attending doctor remain of importance for diagnosis, as detection of bacterial DNA in specimens does not necessarily confirm that the detected organism is the cause of illness [105].

### 4.4. Data Portability and Technology Transition Readiness

Phenotypic results from CBtest are usually in the form of laboratory reports on hard copy printouts or laboratory information system databases of hospitals in middle-income countries, with some countries in this group transitioning to electronic medical records (EMR), including Malaysia [137,138,139]. While hard copy laboratory reports meet the need for result deployment to attending clinicians for patient diagnosis, the information chain stops at this point, and is rarely extracted for further use. In hospitals without EMR, phenotypic results are not recorded electronically, and are therefore seldom readily available for further epidemiologic studies and public health surveillance [140,141].

On the other hand, results from 16SNGS are already in electronic format and allow easy sharing between laboratories. Even though this convenience might not be harvested for day-to-day patient diagnosis, the 16S rRNA sequences of bacterial pathogens could be a useful resource for pathogen surveillance and future epidemiological studies [104]. As the medical sector moves towards digitization and becomes data-driven, new diagnostic microbiology laboratories, including those in middle-income countries, that use 16SNGS for bacterial identification will have higher technology transition readiness [36,41,96]. This happens when diagnostic microbiology ultimately moves towards usage of pathogen whole genome sequence not just for diagnostics, but also as stored information for periodical surveillance, molecular epidemiological studies and public health interventions [84].

## 5. Future Considerations

At the time of writing, many diagnostic microbiology laboratories in middle-income countries, including Malaysia, are still using CBtest as their standard protocol for bacterial identification. Another important type of information required by clinicians for patient treatment (in the case of bacterial infections) will be the pathogen’s antibiotic susceptibility profile. As 16SNGS only identifies the infecting pathogen, antibiotic susceptibility testing (AST) will be required for antibiotic prescription. Even though AST information cannot be obtained via the 16SNGS workflow, the streamlined 16SNGS workflow for identification of unculturable, fastidious and slow-growing bacteria will aid clinicians in providing empirical treatment to their attending patients and to rule out differential diagnosis. Development of rapid AST platforms with real-time monitoring of bacteria growth or inhibition presents the potential for rapid AST results in less than 2 h [142,143]. This process will be faster and phenotypically more accurate compared to curation of antibiotic resistance genomic data from whole genome sequencing workflows. Nevertheless, new technologies will again require substantial costs in procurement, maintenance and training of technical expertise. This will undoubtedly cause delays in technology delivery and deployment in middle-income countries.

**Figure 2:**
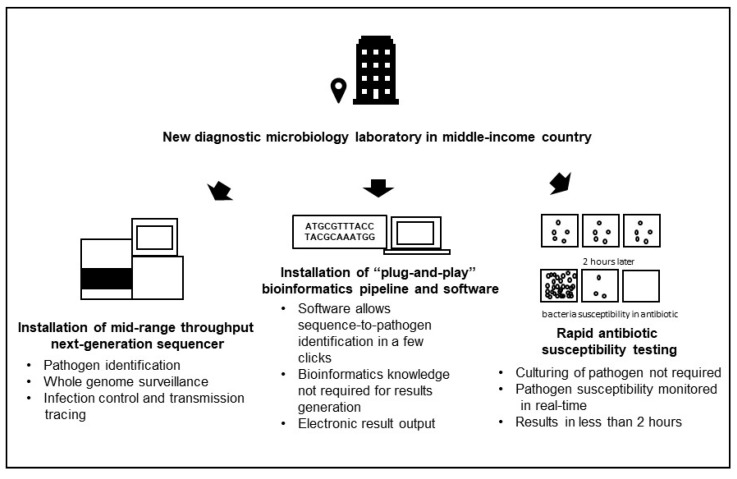
Considerations for new diagnostic microbiology laboratories in middle-income countries utilizing 16SNGS as the main platform for bacterial pathogen detection.

In view of the challenges in using 16SNGS for bacterial identification as described in the earlier sections of this review, diagnostic microbiology laboratories in middle-income countries aiming to use the platform for bacterial identification might consider the following. Firstly, the installation of a mid-range throughput next-generation sequencer which allows for 16SNGS pathogen identification, and also future potential whole genome investigations when the need arises, for example, to investigate nosocomial infection transmission, and for periodical surveillance. Due to cost and technical expertise availability consideration, 16SNGS-based diagnostic microbiology laboratories can be initially established in tier 3 and above hospitals to provide a 16SNGS diagnostic microbiology service to smaller hospitals for cases of unculturable or fastidious bacteria before adoption of the technology by smaller hospitals in the long run. Secondly, a “plug-and-play” bioinformatics pipeline can be used which enables sequence to pathogen identification in a few clicks, without requiring input from a bioinformatician, such as the MYcrobiota and BEPatho applications [144,145]. Thirdly, rapid AST platforms can be used to provide susceptibility testing information for timely antibiotic prescription (Figure 2).

## 6. Concluding Remarks

With the advent of NGS and recent replacement of various serology and biochemical tests with nucleotide-based technologies, 16SNGS has been suggested as a plausible platform for bacterial identification in diagnostic microbiology laboratories. The 16SNGS workflow poses a challenge in terms of technical expertise, funding and validation, especially for middle-income countries. Nevertheless, these challenges could be overcome by market-driven continuous price decline for sequencing, and the commercial availability of bioinformatics applications that allow sequence-to-results generation without bioinformatics knowledge. With its advantages in diagnostic accuracy and streamlined protocols, 16SNGS is poised to play an important and staying role in bacterial identification in diagnostic microbiology laboratories, if it can be deployed successfully in middle-income countries.
